# Microglial Refinement of A-Fiber Projections in the Postnatal Spinal Cord Dorsal Horn Is Required for Normal Maturation of Dynamic Touch

**DOI:** 10.1523/JNEUROSCI.1354-23.2023

**Published:** 2024-01-10

**Authors:** Yajing Xu, Stephanie C. Koch, Alexander Chamessian, Qianru He, Mayya Sundukova, Paul Heppenstall, RuRong Ji, Maria Fitzgerald, Simon Beggs

**Affiliations:** ^1^Neuroscience, Physiology and Pharmacology, UCL, London, WC1E 6BT United Kingdom; ^2^Duke University School of Medicine, Duke University, Durham, North Carolina 27710; ^3^SISSA (International School for Advanced Studies), 34136 Trieste, Italy; ^4^Developmental Neurosciences, UCL Great Ormond Street Institute of Child Health, London, WC1N 1EH United Kingdom

**Keywords:** development, microglia, postnatal, somatosensory, spinal, touch

## Abstract

Sensory systems are shaped in postnatal life by the refinement of synaptic connectivity. In the dorsal horn of the spinal cord, somatosensory circuits undergo postnatal activity-dependent reorganization, including the refinement of primary afferent A-fiber terminals from superficial to deeper spinal dorsal horn laminae which is accompanied by decreases in cutaneous sensitivity. Here, we show in the mouse that microglia, the resident immune cells in the CNS, phagocytose A-fiber terminals in superficial laminae in the first weeks of life. Genetic perturbation of microglial engulfment during the initial postnatal period in either sex prevents the normal process of A-fiber refinement and elimination, resulting in an altered sensitivity of dorsal horn cells to dynamic tactile cutaneous stimulation, and behavioral hypersensitivity to dynamic touch. Thus, functional microglia are necessary for the normal postnatal development of dorsal horn sensory circuits. In the absence of microglial engulfment, superfluous A-fiber projections remain in the dorsal horn, and the balance of sensory connectivity is disrupted, leading to lifelong hypersensitivity to dynamic touch.

## Significance Statement

Dynamic touch is the sensation of movement across the skin, transmitted by mechanosensory A-fibers, the myelinated primary afferents that respond to innocuous mechanical stimulation. The central terminals of these fibers are located in the deep laminae of the sensory spinal cord dorsal horn in the adult. However, in early life, they are widespread and retract from the superficial laminae of the dorsal horn during normal postnatal development. The underlying mechanisms remain unknown. We found that microglia phagocytose superfluous A-fibers, and furthermore, disruption of this process leads to long-term aberrant dynamic touch processing and behavior. Microglia-mediated refinement of A-fibers during the early postnatal period is therefore critical to both normal dorsal horn development and appropriate spatial encoding of dynamic touch.

## Introduction

The neonatal spinal dorsal horn differs substantially from that in adults and undergoes extensive structural and functional reorganization over the postnatal period. One notable change is the termination zone of primary afferent A-fibers, the large myelinated afferents that include many low threshold cutaneous tactile afferents (Abraira and Ginty, 2013). These afferent terminals occupy both superficial and deep laminae of the dorsal horn in neonatal rodents and gradually retract to terminate in deeper laminae III–IV by the end of the third postnatal week where they remain in adulthood (Pignatelli et al., 1989; Fitzgerald et al., 1994; Jennings and Fitzgerald, 1996). This retraction of A-fiber terminals is accompanied by a reduction in dorsal horn cell mechanosensitive receptive field sizes on the skin, as well as a decline in tactile sensitivity and an increase in reflex behavior precision, with similar changes in somatosensory behavior observed in human infants (Fitzgerald, 1985, 2015; Fitzgerald et al., 1988). This suggests that structural refinement in the dorsal horn likely underlies postnatal somatosensory behavioral maturation.

The process of A-fiber terminal retraction in the first weeks of life is activity dependent: blocking neuronal input through spinal NMDAR inhibitors or increasing the noise of neuronal input through random vibration to the skin over extended period prevents the normal retraction of A-fibers (Beggs et al., 2002; Granmo et al., 2008). However, the exact mechanism underlying the retraction of A-fiber terminals is not known. Microglia cells, the major phagocytes in the CNS, have been shown to remove superfluous neurons by driving apoptosis, removing apoptotic cells, and phagocytosing synapses and neurites during postnatal refinement (Salter and Stevens, 2017). To date, such studies have been largely restricted to the brain (Paolicelli et al., 2011; Schafer et al., 2012; Lui et al., 2016; Gunner et al., 2019; Milinkeviciute et al., 2019), with two studies reporting a role of microglia in the postnatal development of spinal cord ventral horn motor circuits (Vainchtein et al., 2018; Vukojicic et al., 2019). Whether microglia are also involved in the maturation of somatosensory circuits in the dorsal horn is not known. Here we hypothesize that the retraction of A-fibers from superficial laminae in the postnatal period is driven by microglia which prune A-fiber terminals in the dorsal horn as part of normal postnatal development of spinal sensory circuits.

Microglia undergo postnatal maturation during which they not only change in density and morphology but also alter their transcriptional and functional identity (Zusso et al., 2012; Matcovitch-Natan et al., 2016). Brain microglia show particularly high expression of lysosome associated genes at P4/5 suggesting a specialized role of microglial phagocytosis during development (Hammond et al., 2019). In addition, microglia exhibit spatial heterogeneity, as they populate different brain regions at different rates postnatally, and express distinct local genetic profiles and phenotype in adulthood (Schwarz et al., 2012; De Biase et al., 2017; Ayata et al., 2018). To date most research has focused on the brain and relatively little is known about spinal cord microglia.

Here we have mapped A-fiber terminal development in the spinal dorsal horn using *Vglut1* reporter mice combined with microglial immunolabeling to quantify engulfment of neuronal and synaptic elements across early postnatal life. To test whether normal microglial phagocytosis is required for the pruning and elimination of superfluous A-fiber terminals, we blocked microglial phagocytosis during the first postnatal week using a tamoxifen inducible Cre-mediated deletion of the *Tmem16f* gene in microglia. The effect of this upon A-fiber pruning, maturation of dorsal horn synaptic connections, and dynamic and static tactile sensory processing was assessed. The results show that microglia prune A-fiber terminals in the developing spinal dorsal horn and that this postnatal microglial refinement of A-fiber terminals is required for normal somatosensory maturation.

## Materials and Methods

### Animals

Transgenic mice on C57BL/6J background of both sexes were used in all other experiments.

Experiments used the following transgenic mouse lines:

1. Ai9/Rosa26-CAG::loxP-STOP-loxP-tdTomato-WPRE (Jackson Laboratory RRID:IMSR_JAX:007909 and RRID:IMSR_JAX:007905)
2. *Slc17a7*-IRES2-Cre (Jackson Laboratory RRID:IMSR_JAX:023527)
3. *Cx3cr1*-CreERT2-YFP (Jackson Laboratory RRID:IMSR_JAX:021160)
4. *Thy1*-EGFP-M (Jackson Laboratory RRID:IMSR_JAX:007788)
5. *Tmem16f*-floxed (flx) animals (Batti et al., 2016)

For visualization of A-fibers, *Slc17a7*-IRES2-Cre (*Vglut1*-Cre) males (JAX stock number 023527) were crossed with Ai9 females (JAX stock number 007909) to obtain animals that expressed the tdTomato fluorophore under the *Vglut1* promoter (*Vglut1^Cre/+^*; *R26^LSL-Ai9/+^*).

To generate tamoxifen inducible microglia-specific *Tmem16f* knock-out mice (*Tmem16f* cKO), Cx3cr1-CreER-YFP (JAX stock number 021160) mice were crossed to *Tmem16f*-flx animals (generated by P. Heppenstall, Batti et al., 2016), as well as Ai9 (JAX stock number 007905) and *Thy1*-EGFP-M (JAX stock number 007788).

Experimental animals were heterozygous for *Cx3cr1*-CreER-YFP (^CreER/+^), homozygous for mutant conditional allele *Tmem16f*-flx (^fl/fl^), and carrying Ai9 (*R26^LSL-Ai9^*) and *Thy1*-eGFP (^eGFP^) alleles (zygocity was not determined for *R26^LSL-Ai9^* and *Thy1^eGFP^*). This produced the following genotype: *Cx3cr1^CreER/+^*, *Tmem16f^fl/fl^*, *R26^LSL-Ai9^*, and *Thy1^eGFP^*. Control animals were homozygous for the wild-type *Tmem16f* allele: *Cx3cr1^CreER/+^*, *Tmem16f^+/+^*, *R26^LSL-Ai9^*, and *Thy1^eGFP^*.

To control for off-target effects of Cre expression and tamoxifen administration, cKO mice were *Tmem16f^fl/fl^* and controls *Tmem16f^+/+^* (*Cx3cr1^CreER/+^*, *Tmem16f^fl/fl^*, *R26^LSL-Ai9^*, *Thy1^eGFP^* and *Cx3cr1^CreER/+^*, *Tmem16f^+/+^*, *R26^LSL-Ai9^*, and *Thy1^eGFP^*, respectively). Both groups received 4-hydroxytamoxifen (4-HT) daily from P1 to P3 and were assessed at 3–4 months old.

Both females and males were used. No sex differences were expected, and animals of both sexes were pooled together for analysis, but data points are presented as black (female) or red/magenta/blue (male) to indicate the sexes. Numbers of animals used for each experiment are indicated in the figures. For a table with detailed species, ages, sexes, and numbers of animals used in each experiment, please see Table 1. All procedures were carried out in accordance with the guidelines of the UK Animals (Scientific Procedures) Act 1986 and subsequent amendments.

**Table 1.** Complete statistical analysis

Animals	*N*-values (f, m)	Factors/comparisons	Mean difference	ci_width	ci_lower_limit	ci_upper_limit	Permutation *t* test *p*-value	Significance test	Factors and interactions	Df, *F*, *t*, *z*, *R* value	*p*-value	Dunnett's *post hoc* adjusted *p*-value
Vglut1-Cre::Ai9 mice P0, P3, P7, P10, P17, P28	7 (3, 4), 8 (4, 4), 9 (4, 5) 8 (5, 3), 12 (6, 6), 12 (6, 6)	L1–2: P0 versus P3	0.482228929	95%	−23.5239025	24.35441643	0.9724	One-way ANOVA	Age	*F* (5, 50) = 22.85	*p* < 0.0001	0.9999
		L1–2: P0 versus P7	52.19286032	95%	23.71181302	79.47354032	0.0098					0.0021
		L1–2: P0 versus P10	117.1632364	95%	92.25760036	137.6065632	0					0.0001
		L1–2: P0 versus P17	2.519137262	95%	−15.066162	18.37541619	0.79					0.9997
		L1–2: P0 versus P28	31.84151893	95%	11.41666619	49.1574	0.0098					0.075
	7 (3, 4), 8 (4, 4), 9 (4, 5) 8 (5, 3), 12 (6, 6), 12 (6, 6)	L3–4: P0 versus P3	−66.6795988	95%	−121.068375	−18.1410261	0.034	One-way ANOVA	Age	*F* (5, 50) = 48.1	*p* < 0.0001	0.0043
		L3–4: P0 versus P7	−19.4751778	95%	−67.5350457	21.66349905	0.4192					0.7377
		L3–4: P0 versus P10	136.01636	95%	82.89459464	184.4138782	0.0006					0.0001
		L3–4: P0 versus P17	−100.850303	95%	−145.398786	−62.4165912	0					0.0001
		L3–4: P0 versus P28	−74.9477658	95%	−119.96932	−36.6079167	0.0002					0.0004
	7 (3, 4), 8 (4, 4), 9 (4, 5), 5 (1, 4), 12 (6, 6), 12 (6, 6)	L1–2: P0 versus P3	1.459834446	95%	−6.85984861	6.288613804	0.6782	One-way ANOVA	Age	*F* (5, 47) = 4.804	*p* = 0.0013	0.5443
		L1–2: P0 versus P7	4.047300016	95%	−4.10881754	8.177807683	0.1804					0.0886
		L1–2: P0 versus P10	−3.28224903	95%	−10.758767	1.107532314	0.4388					0.2309
		L1–2: P0 versus P17	−3.68936376	95%	−11.5686392	−0.10200417	0.111					0.0996
		L1–2: P0 versus P28	−4.11252835	95%	−11.7853885	−0.38255051	0.1072					0.0674
	7 (3, 4), 8 (4, 4), 9 (4, 5), 5 (1, 4), 12 (6, 6), 12 (6, 6)	L3–4: P0 versus P3	−13.4683884	95%	−31.9728706	2.150880357	0.1654	One-way ANOVA	Age	*F* (5, 47) = 11.78	*p* < 0.0001	0.0392
		L3–4: P0 versus P7	11.03193683	95%	−7.37068889	26.33391492	0.221					0.0808
		L3–4: P0 versus P10	−7.05416229	95%	−24.5196677	6.615074	0.5076					0.3312
		L3–4: P0 versus P17	−24.5478671	95%	−40.8175379	−11.2714589	0.0008					0.0001
		L3–4: P0 versus P28	−21.4929715	95%	−37.9345753	−7.922356	0.0042					0.0006
Cx3cr1 Cre:: Tmem16f flx:: Thy1-GFP::Ai9 mice, adults	8 (4, 4), 8 (4, 4)	flx−/− versus flx+/+	799.276125	95%	450.0915	1,136.345125	0.0006	Welch's *t* test		*t* = 4.201 degrees of freedom = 13.13	0.001	
								*F* test		*F* (7, 7) = 1.694	0.5033	
	8 (4, 4), 8 (4, 4)	flx−/− versus flx+/+	533.6704929	95%	356.11875	695.4979929	0.0008	Welch's *t* test		*t* = 5.610 degrees of freedom = 12.09	0.0001	
								*F* test		*F* (7, 6) = 2.377	0.3112	
	8 (4, 4), 8 (4, 4)	flx−/− versus flx+/+	−168.3105	95%	−562.60575	253.437875	0.4566	Welch's *t* test		*t* = 0.7542 degrees of freedom = 10.83	0.4668	
								*F* test		*F* (7, 7) = 3.357	0.1325	
	8 (4, 4), 8 (4, 4)	flx−/− versus flx+/+	−159.18725	95%	−416.4165	148.87525	0.3194	Welch's *t* test		*t* = 1.028 degrees of freedom = 12.91	0.3227	
								*F* test		*F* (7, 7) = 1.820	0.4477	
	8 (4, 4), 8 (4, 4)	flx−/− versus flx+/+	201.3333375	95.00%	110.9028125	292.0000125	0.0012	Welch's *t* test		*t* = 4.115 degrees of freedom = 7.9	0.0035	
								*F* test		*F* (7, 7) = 15.5	0.0018	
	8 (4, 4), 8 (4, 4)	flx−/− versus flx+/+	56.6111125	95.00%	−13.8055625	118.416675	0.1352	Welch's *t* test		*t* = 1.546 degrees of freedom = 13.75	0.1448	
								*F* test		*F* (7,7) = 1.312	0.7294	
	4 (3, 1), 4 (3, 1)	flx−/− versus flx+/+						Welch's *t* test		*t* = 0.102 degrees of freedom = 5.996	0.9221	
								*F* test		*F*_(3,3)_ = 1.051	0.9681	
	16 (5, 11), 26 (11, 15)	flx−/− versus flx+/+	0.836538462	95%	0.072115385	1.572115385	0.0132	Mann−Whitney test		Mann−Whitney *U* = 136.5	0.0543	
	flx−/−: 16 (5, 11) all, Flx+/+: 26 (11, 15) all	flx−/− versus flx+/+	0.115277778	95%	−0.38981482	0.649074074	0.6484	Two-way ANOVA	Interaction	*F* (4, 200) = 0.6123	*p* = 0.6542	
									Row: vF strength	*F* (4, 200) = 172.2	*p* < 0.0001	
									Column: genotype	*F* (1, 200) = 0.7813	*p* = 0.3778	
	22 (10, 12), 23 (13, 10)	flx−/− versus flx+/+	13.69027147	95%	3.314773298	27.19848043	0.0274	Welch's *t* test		*t* = 2.243 degrees of freedom = 31.42	0.0321	
								*F* test		*F*_(22,21)_ = 4.607	0.0009	
	22 (10, 12), 23 (13, 10)	flx−/− versus flx+/+	−11.3543475	95%	−19.2225949	−4.45579651	0.002	Welch's *t* test		*t* = 2.933 degrees of freedom = 38.59	0.0056	
								*F* test		*F* (21, 22) = 1.835	0.1654	
	flx−/−: 22 (10, 12) all, flx+/+: 23 (13, 10) all	Interaction	−6.41391901	95%	−8.509839	−4.5015693	0	Two-way ANOVA		*F* (9, 424) = 6.095	*p* < 0.0001	
		Row: vF strength								*F* (9, 424) = 39.91	*p* < 0.0001	
		Column: genotype								*F* (1, 424) = 72.68	*p* < 0.0001	
	8 (4, 4), 5 (2, 3)	flx−/− versus flx+/+	5.53078865	95.00%	−15.9093662	40.43096265	0.6754	Welch's *t* test		*t* = 0.3727 degrees of freedom = 7.705	0.7194	
								*F* test		*F* (4, 7) = 1.317	0.7023	
	8 (4, 4), 5 (2, 3)	flx−/− versus flx+/+	8.61547675	95.00%	−5.7761925	22.72381075	0.2978	Welch's *t* test		*t* = 1.054 degrees of freedom = 7.233	0.3259	
								*F* test		*F* (4, 7) = 1.536	0.5811	
	8 (4, 4), 5 (2, 3)	Interaction	2.742708278	95.00%	−0.0737499	6.247083232	0.0602	Two-way ANOVA	Interaction	*F* (9, 110) = 0.5658	*p* = 0.8224	
		Row: vF strength							Row: vF strength	*F* (9, 110) = 17.25	*p* < 0.0001	
		Column: genotype							Column: genotype	*F* (1, 110) = 7.014	*p* = 0.0093	

Estimation stats permutation test values alongside significance testing and *p*-values.

### Drugs

4-HT was dissolved at 1 mg/ml in corn oil, and 50 μl was injected intragastrically per pup daily on 3 consecutive days from P1–3, following a previously described protocol (Pitulescu et al., 2010). Both control and experimental animals (*Tmem16f^+/+^* and *Tmem16f^fl/fl^*, respectively, see Animals section above) received 4-HT injections to control for any effects of 4-HT itself. The dam was given a protein enriched diet a few days before and following delivery to aid milk production and pup survival.

### Immunohistochemistry

Animals were overdosed with pentobarbital and transcardially perfused with saline followed by ice-cold 10% formalin. The sciatic nerve was exposed and traced to locate L4 and L5 dorsal root ganglia (DRG) and the corresponding region of the lumbar spinal cord was dissected and post-fixed in 10% formalin overnight, followed by immersion in 30% sucrose until they sank. The 50 μm free-floating spinal cord sections were cut on the microtome with every second section collected.

Tissue sections were washed 3 × 10 min in PBS and then incubated in blocking solution (10% donkey serum, 0.2% Triton X-100 in PBS) for 2.5 h at room temperature. The sections were then incubated with primary antibodies at 4°C overnight followed by secondary antibodies at room temperature for 2 h, both diluted in 3% blocking solution (3% donkey serum, 0.2% Triton X-100 in PBS) (for the list of antibodies and their respective concentrations used, see Table 2). Samples were mounted in Fluoromount Aqueous Mounting Medium (Sigma) or ProLong Diamond Antifade Mountant (Thermo Fischer), if the tissue contained endogenous fluorophores.

**Table 2.** List of antibodies used

	Company	Cat#	Concentration	RRID
Primary antibodies				
Mouse anti-CD68	BIO-RAD	MCA341R	1:500	RRID:AB_2291300
Rabbit anti-CD68	Abcam	Ab125212	1:500	RRID:AB_10975465
Rabbit anti-iba1	Wako	019-19741	1:1,000	RRID:AB_839504
Goat anti-iba1	Abcam	Ab5076	1:1,000	RRID:AB_2224402
Mouse anti-NeuN	Millipore	MAB377	1:2,000	RRID:AB_2298772
Mouse anti-VGAT	Synaptic Systems	131011	1:2,000	RRID:AB_2619818
Rabbit anti-VGAT	Synaptic Systems	131002	1:2,000	RRID:AB_887871
Guinea pig anti-VGluT2	Millipore	AB2251	1:2,000	RRID:AB_1587626
Mouse anti-synaptophysin	Abcam	Ab809	1:2,000	RRID:AB_2058440
Guinea pig anti-synaptophysin	Synaptic Systems	101004	1:2,000	RRID:AB_1210382
Secondary antibodies				
Donkey anti-goat Cy2	Jackson Immunoresearch	705-225-147	1:500	RRID:AB_2307341
Donkey anti-goat Cy3	Jackson Immunoresearch	705-165-147	1:500	RRID:AB_2307351
Donkey anti-goatCy5	Jackson Immunoresearch	705-175-147	1:500	RRID:AB_2340415
Donkey anti-goat Alexa 488	Jackson Immunoresearch	705-545-003	1:500	RRID:AB_2340428
Donkey anti-guinea pig Cy3	Jackson Immunoresearch	706-165-148	1:500	RRID:AB_2340460
Donkey anti-guinea pig Alexa 647	Millipore	AP193SA6	1:500	RRID:AB_2629452
Donkey anti-mouse Cy2	Jackson Immunoresearch	715-225-150	1:500	RRID:AB_2340826
Donkey anti-mouse Cy3	Jackson Immunoresearch	715-165-150	1:500	RRID:AB_2340813
Donkey anti-mouse Cy5	Jackson Immunoresearch	715-175-150	1:500	RRID:AB_2340819
Donkey anti-rabbit Cy2	Jackson Immunoresearch	711-225-152	1:500	RRID:AB_2340612
Donkey anti-rabbit Alexa 594	Jackson Immunoresearch	711-585-152	1:500	RRID:AB_2340621
Donkey anti-rabbit Alexa 647	Millipore	AP182SA6	1:500	
Horse anti-rabbit biotinylated	Vector	BA11-0	1:500	
Streptavidin-594	Life Technologies	S3356	1:500	

### Image acquisition and analysis

Confocal z-stacks were taken with a Zeiss LSM880 confocal microscope or a Yokogawa CSU22 spinning disk microscope using a 20× water immersion objective (NA 1.0) for imaging of A-fibers, 20× air objective (NA 0.8) for imaging of IB4, and 63× oil immersion objective (NA 1.4) for imaging of synaptic markers followed by analysis in Fiji software. Details on microscope settings can be found in the metadata of example images online at https://github.com/Yajing826/A-fibre-engulfment and raw data files at the EBI Bioimage Archive under the accession number S-BSST609: https://www.ebi.ac.uk/biostudies/bioimages/studies/S-BSST609. Only intact sections with an even stain were analyzed, and at least 6 sections were imaged and analyzed per animal to reduce variability for all figures.

A-fiber engulfment by microglia and synapse density were analyzed with automated batch processing in Fiji using the 3D-ROI manager plugin and custom written macros (Linkert et al., 2010; Schindelin et al., 2012; Schneider et al., 2012; Ollion et al., 2013). For A-fiber engulfment, each of the channels containing staining for microglia, lysosomes, or A-fibers was binarized, and the volume of their overlap measured. For synapse density measures, the channel containing synaptic stain was binarized and segmented, following which volume and object numbers were recorded. Macro-scripts for the automated analysis are available online at https://github.com/Yajing826/A-fibre-engulfment

### Behavior

Behavioral testing was carried out on adult mice of both sexes between 3 and 4 months old, with the experimenter blinded to animal genotype/treatment. Animals were placed on a mesh platform (Ugo Basile) within individual transparent plastic chambers (6 cm × 6 cm × 12 cm) for sensory testing of the plantar surface of the hindpaw. Habituation and testing happened over 5 consecutive days. Animals were habituated to the testing environment for 1 h per day on the first 2 d within individual plexicon chambers on the mesh platform. On the remaining days, animals were habituated for 30 min before being tested on each day. The dynamic touch response and static touch thresholds were determined on the third day, while the repeated vF response testing was spread over the remaining 2 d to avoid sensitization. A number of withdrawal reflexes were scored in each case, where only a rapid paw lifting was scored as a reflex. Animals were allowed to rest at least 20 s between each stimulus. For the dynamic touch response, a fine brush (Pro Arte, series 007, size 2) was moved over the plantar surface of the hindpaw from the heel to toe over a 2 s period (Duan et al., 2014). This was repeated five times, and the number of withdrawal reflexes out of five was recorded. The vF threshold was assessed using the simplified up and down method (Bonin et al., 2014). The filaments were aimed at the region between the footpads. Force was applied until the filament bent and held in place for 2 s.

To generate a response curve to vF stimulation, a repeated vF response was recorded by applying each of filaments number 3–7 (0.04–0.6 g) five times on the plantar surface, directed at the region between the footpads. The sequence of vF filaments was randomized. Number of withdrawal reflexes out of five times was recorded.

### In vivo extracellular recording

Animals subjected to behavioral testing were reused in electrophysiological recordings. An experimenter was blinded to animal genotype/treatment. All recordings were performed on adult mice (3–4 months old) of both sexes in the deep dorsal horn. Cells were recorded between 200 and 550 μm depth from the surface of the spinal cord. 3–5 mice were used per sex and treatment group. All experiments were carried out by the same experimenter to ensure consistency.

### Animal preparation

Mice were anaesthetized with intraperitoneal urethane injection (10% in saline, 1.5 g/kg). Approximately 100 μl of 0.6 g/ml atropine and 200 μl of saline were injected subcutaneously to, respectively, counteract the mucus-driving side effect of urethane and to prevent dehydration. The animal was constantly monitored for depth of anesthesia throughout the experiment and supplemented with 50 μl (5 μg) urethane as needed. Approximately 200 μl of saline was supplemented every 2 h. The body temperature of the animal was kept close to 37°C with a heating pad throughout.

After cessation of reflexes, a tracheotomy was performed and a short plastic tube of approximately 1 cm inserted to aid free breathing of the animal. The animal was then transferred onto a stereotactic frame and fixed with ear and hip bars. A laminectomy was carried out at vertebral level T13-L1 which corresponds to the spinal segments L4–L5 underneath. The spinal column was clamped for stability, the dura was removed, and the exposed spinal cord was covered with mineral oil to prevent drying.

### Single unit extracellular recordings

A carbon micro-electrode (Carbostar-1, Kation Scientific) was lowered with a motorized manipulator (Scientifica) into the exposed spinal cord at a constant speed. A reference electrode was inserted into the back muscle close to the laminectomy for differential recording. Recorded neural activity was amplified 2000 times and filtered for signals between 1 kHz and 10 kHz (NL104 amplifier and NL125/6 bandpass filter modules from NeuroLog Digitimer). The signal was sampled at 20 kHz and digitized using PowerLab 4/30 (ADInstruments). The trace was recorded and analyzed in the software LabChart 7 (ADInstruments).

To isolate single neurons, the plantar surface of the animal's hindpaw was gently continuously stroked as a search signal, while the electrode was lowered through the dorsal horn of the spinal cord. Once a cell was identified from consistent spike amplitude, the dynamic touch receptive field of the cell was mapped out by a fine brush (Pro Arte, series 202, size 1, brush tip cut short to 7 mm length × 1 mm width).

Spontaneous activity was recorded for 10 min before and 5 min after stimulation. To record dynamic and static [von-Frey (vF) fiber] evoked activity from single neurons, each stimulus was manually applied for 2 s within the receptive field of the cell and repeated three times, with a minimum of 10 s interval in between (vF filament strength were as follows: 1 = 0.008 g, 2 = 0.02 g, 3 = 0.04, 4 = 0.07 g, 5 = 0.16 g, 6 = 0.40 g, 7 = 0.60 g, 8 = 1 g, 9 = 1.7, 10 = 2 g).

Cells with very high spontaneous firing rates where evoked activity could not be clearly distinguished from spontaneous activity were not recorded. Only wide dynamic range neurons responding both to dynamic (brush) and static (von Frey hair and pinch stimulation) were recorded. Animals were euthanized at the end of the experiment and the spinal cord was collected in neutral buffered 10% formalin (overnight) followed by 30% sucrose solution for reuse in immunohistochemistry.

### Analysis and cell type categorization

Analysis was carried out in the LabChart 7 software (ADInstruments). For spontaneous activity, firing rate was analyzed over a 10 min window prior to applying any stimuli. Firing rate for evoked responses were analyzed over the first second of the stimulus duration and averaged over three trials.

Cells were divided into adapting and nonadapting groups based on their firing properties toward static touch, that is a vF threshold stimulus (Lee et al., 2019). This vF threshold was defined as the first vF filament that evokes a firing rate of 10 Hz or more. The response within the first second of vF application was analyzed to calculate an adaptive ratio *R*, which was defined as

$$R=\frac{\mathrm{Number}\mathrm{of}\mathrm{pikes}\mathrm{fired}\mathrm{between}0.5\text{-}1s}{\mathrm{Number}\mathrm{of}\mathrm{spikes}\mathrm{fired}\mathrm{between}0\text{-}0.5s}.$$

If a cell adapts rapidly to stimulation, one would expect *R* to be close to zero, as barely any spikes should be fired between 0.5 and 1 s; however, if a cell is nonadapting and firing continuously, one would expect *R* to be close to 1. To decide the boundary between adapting and nonadapting cells we used *k*-means cluster analysis, which sorted the values into two groups that is equivalent to a boundary at *R* = 0.33. For the *k*-means clustering we included cells from a previous experiment.

### Statistical analysis

Estimation statistics were used throughout the manuscript. Graphs are plotted with effect size and 95% confidence intervals adjacent to all data points. Estimation statistics were calculated on estimationstats.com (Ho et al., 2019) using 5000 samples of bias-corrected and accelerated bootstrapping. Data are presented as mean ± SD in all figures and estimation statistics are presented as adjacent Gardner–Altman estimation plots. The effect size is presented as 95% CI of the mean difference which is plotted as a dot on the background of its probability distribution, with the 95% confidence interval indicated by the ends of the error bar. All values in text and figures are given with two decimals or rounded to two significant figures. Sample numbers (*n*) are as indicated in figures.

Additionally, conventional null-hypothesis significance testing was carried out on estimationstats.com and GraphPad Prism 6 for all comparisons (significance level was set at *α* = 0.05). For a comprehensive list with exact statistical values and analyses, see Table 1.

### Data availability

All data generated or analyzed during this study are included in the manuscript and supporting files. Source files for all data are available on the EBI Bioimage Archive (https://www.ebi.ac.uk/bioimage-archive/) under the accession number S-BSST609: https://www.ebi.ac.uk/biostudies/bioimages/studies/S-BSST609.

Macro-scripts for automated analysis in Fiji are available online at https://github.com/Yajing826/A-fibre-engulfment.

## Results

### Spinal microglia phagocytose A-fiber terminals during early postnatal period

**Figure 1.**
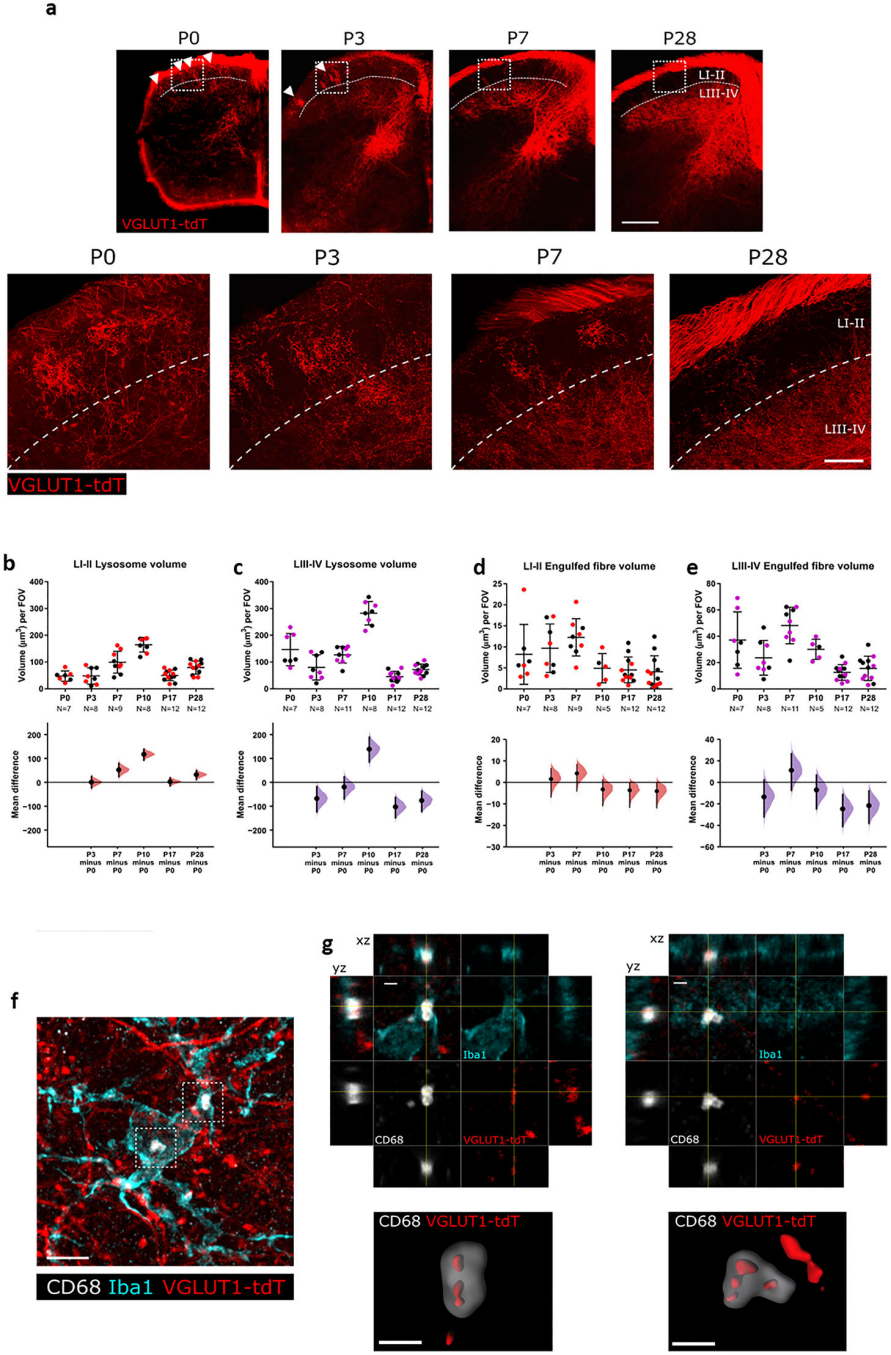
Spinal dorsal horn microglia-engulfed A-fibers during normal postnatal development. ***a***, VGluT1-tdTomato (red) expression in spinal laminae I–II changes with postnatal age (P0, 3, 7, and 28). White boxes indicate regions shown in higher power images below. Dashed white line indicates the lamina (L) II/III border. Scale bar = 50 μm. ***b***, Microglial lysosome volume peaks at P10 and decreases thereafter for LI–II. P3 versus P0, mean difference 0.48 (95% CI 23.52, 24.35); P7 versus P0, mean difference 52.19 (95% CI 23.71, 79.47); P10 versus P0, mean difference 117.16 (95% CI 92.26, 137.61); P17 versus P0, mean difference 2.52 (95% CI −15.07, 18.38); P28 versus P0, mean difference 31.84 (95% CI 11.42, 49.16). Field of view (FOV) = 245 μm × 65 μm. ***c***, Microglial lysosome volume peaks at P10 and decreases thereafter for LIII–IV. P3 versus P0, mean difference −66.67 (95% CI −121.07, −18.14); P7 versus P0, mean difference −19.48 (95% CI −67.54, 21.66); P10 versus P0, mean difference 136.02 (95% CI 82.89, 184.41); P17 versus P0, mean difference −100.85 (95% CI −145.40, −62.42); P28 versus P0, mean difference −74.95 (95% CI −119.97, −36.61). Field of view (FOV) = 245 μm × 65 μm. ***d***, Engulfed fiber volume peaks at P7 and decreases thereafter for LI–II. P3 versus P0, mean difference 1.46 (95% CI −6.86, 6.29); P7 versus P0, mean difference 4.05 (95% CI −4.11, 8.18); P10 versus P0, mean difference −3.28 (95% CI −10.76, 1.11); P17 versus P0, mean difference −3.69 (95% CI −11.57, −0.10); P28 versus P0, mean difference −4.11 (95% CI −11.79, −0.38). Field of view (FOV) = 245 μm × 65 μm. ***e***, Engulfed fiber volume peaks at P7 and decreases thereafter for LIII–IV. P3 versus P0, mean difference −13.47 (95% CI −31.97, 2.15); P7 versus P0, mean difference 11.03 (95% CI −7.37, 26.33); P10 versus P0: mean difference −7.05 (95% CI −24.52, 6.62); P17 versus P0, mean difference −24.55 (95% CI −40.82, −11.27); P28 versus P0, mean difference −21.49 (95% CI −37.93, −7.92). Field of view (FOV) = 245 μm × 65 μm. Numbers of animals (*n*) indicated each age. Black and colored data points indicate females and males, respectively. ***f***, Representative *z*-projected super-resolution image of A-fiber engulfment by microglia within the cell body. White inset box show location of higher magnification panels in g. Scale bar = 5 μm. ***g***, High magnification images of microglial A-fiber engulfment in b stained for microglia (Iba1, cyan), microglial lysosomes (CD68, white) and endogenously fluorescent A-fibers (VGluT1-tdT, red). Cross-hairs show position of the *xz* and *yz* side-view panels. Bottom panels show surface rendering of the super-resolution image revealing pieces of tdT labeled fibers engulfed inside the lysosome. Scale bar = 1 μm.

In the developing spinal cord, innocuous touch-encoding A-fibers initially project throughout the dorsoventral extent of the dorsal horn and refine over the first few postnatal weeks to terminate in deeper laminae, segregated from the more superficial terminals of noxious-encoding C-fibers (Beggs et al., 2002). We confirmed this using a transgenic reporter mouse in which tdTomato is expressed in vesicular glutamate transporter 1 (*Vglut1*)-expressing neurons (VGluT1-tdT), a subpopulation of large myelinated sensory neurons with features consistent with Aβ-low threshold mechanoreceptors (LTMRs) (Chamessian et al., 2019). Characteristic A-fiber flame-shaped arbors were present in lamina I–II until P7 (Fig. 1*a*), after which they dissipate and are no longer detectable by P28, consistent with previous findings (Beggs et al., 2002).

We hypothesized that this refinement of superficial axon terminals involves microglial phagocytosis. To test this, we used immunohistochemistry to quantify the phagocytic activity of spinal dorsal horn microglia using the lysosomal associated molecular marker CD68. Microglial lysosomal volume increased in both superficial (I–II) and deeper (III–IV) spinal cord laminae over the postnatal period, peaking at P10 and declining thereafter (Fig. 1*b*,*c*) [Lamina (L)I–II P10 versus P0 unpaired mean difference 117.16 µm^3^ (95% CI 92.26, 137.61); LIII–LIV unpaired mean difference 136.02 µm^3^ (95% CI 82.89, 184.41)]. We next asked whether the peak in microglial lysosomal volume, indicative of phagocytic function (Solé-Domènech et al., 2016), is associated with the refinement of afferent connectivity in the dorsal horn through the removal of superfluous A-fiber terminals. Engulfment of A-fibers was measured by quantifying the colocalization of VGluT1-tdT fluorescence with CD68-positive lysosomes within Iba1-labeled microglia cells (Fig. 1*d*,*e*; example shown in *g*,*h*).

Consistent with the postnatal increase in microglial lysosome volume, the volume of engulfed putative A-fiber terminals (as indicated by intralysosomal tdTomato fluorescence) was elevated during the first postnatal week, peaking at P7 and decreasing thereafter in both laminae (L) I–II, [unpaired mean difference P28 vs P0 −4.11 µm^3^ (95% CI −11.79, −0.38)] and laminae III–IV [unpaired mean difference P28 vs P0 −21.49 µm^3^ (95% CI −37.93, −7.92)].

### Microglial activity is required for normal A-fiber pruning in laminae I–II

Next, we asked whether microglial function is necessary for A-fiber engulfment. The phospholipid scramblase TMEM16F is required for microglial phagocytic activity (Batti et al., 2016). To address this question, we disrupted microglial function postnatally using a Cre-inducible conditional knock out of *Tmem16f* in microglia (Batti et al., 2016) and examined the structural, functional, and behavioral consequences in adult animals. A *R26^LSL-Ai9^* reporter line was used to label *Cxc3cr1^Cre^*-expressing microglial cells (Madisen et al., 2010; Parkhurst et al., 2013), and a *Thy1^eGFP^* allele was used to identify A-fibers (Feng et al., 2000; Taylor-Clark et al., 2015).

### VGluT1+ terminals are increased in superficial, but not deep laminae, in adult microglial *Tmem16f* cKO mice

**Figure 2.**
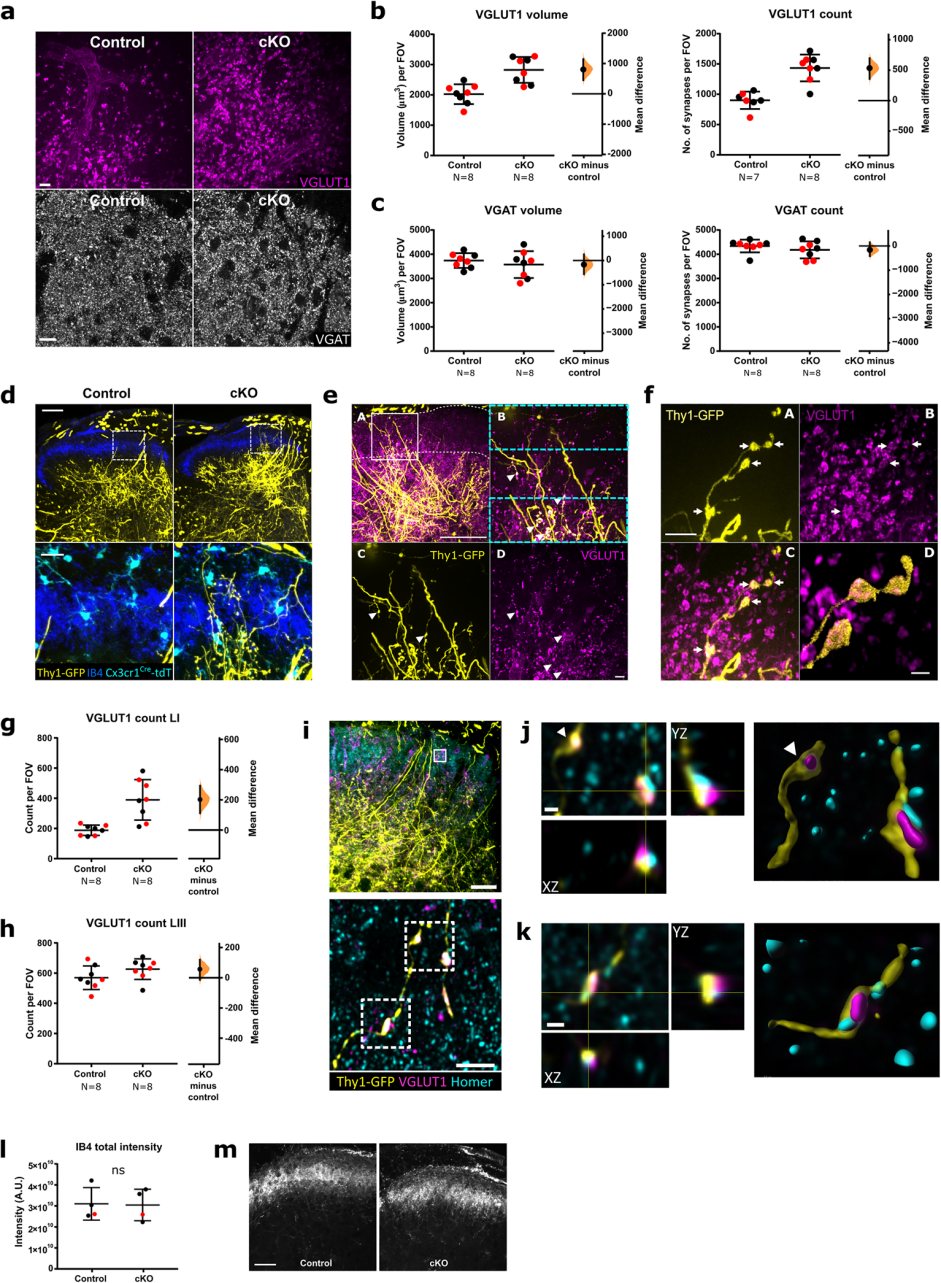
A-fiber terminals are retained in the superficial dorsal horn of adult animals following neonatal *Tmem16f* microglial knock-out (*Tmem16f* cKO). ***a***, Representative images of VGluT1 and VGAT puncta from the superficial spinal dorsal horn of adult *Tmem16f* cKO and control animals. Field of view (FOV) = 94 μm × 94 μm (VGluT1), 96 μm × 96 μm (VGAT). Scale bars = 10 μm. ***b***, VGluT1 puncta volume and count were both increased in adult *Tmem16f* cKO animals compared to controls. Control versus cKO volume: mean difference 799.28 µm^3^ [95% CI 450.09, 1,136.35]; control versus cKO count: mean difference 533.67 (95% CI 356.12, 695.50). ***c***, VGAT puncta volume and count were not significantly different between adult *Tmem16f* cKO animals and controls. Control versus cKO volume: mean difference −168.3105 µm^3^ (95% CI −562.61, 253.44); control versus cKO count: mean difference −159.19 (95% CI −416.42, 148.88). ***d***, Thy1-GFP-labeled A-fibers (yellow) are present in the superficial dorsal horn (delimited by IB4, blue) of adult cKO mice, but not in adult controls. tdTomato-labeled microglia (cyan) are present in both cKO and control animals. Lower panels show high power images of the boxed areas in the upper panels. Scale bar = 100 μm in the top panel and 20 μm in the lower panel. ***e***, Thy1-GFP-labeled A-fibers (yellow) in the superficial dorsal horn laminae of adult *Tmem16f* cKO mice express VGluT1 (magenta). (***A***) Low magnification image showing superficially-projecting Thy1-GFP-labeled fibers. White box indicates where images were taken for analysis in ***a–c***. (***B***) High magnification of boxed region in ***A***. Cyan boxes indicate the cropped areas used for analysis in ***g*,*h***. (***C***) Thy1-GFP labeling. (***D***) VGluT1 immunoreactivity. Scale bar in *A* = 100 µm. Scale bar in *D* = 10 µm. ***f***, (***A*–*D***) High-magnification examples for VGluT1-expression (magenta) in Thy1-GFP-labeled A-fibers (yellow) in adult *Tmem16f* cKO mice. Scale bar in *A* = 20 μm, scale bar in *D* = 5 μm. ***g*,*h***, VGluT1 count in adult *Tmem16f* cKO and control animals in superficial lamina I (***g***) and deep lamina III (***h***). LI VGluT1 count mean difference 201.33 (95.00% CI 110.90, 292.00); LIII VGluT1 count mean difference 56.61 (95.00% CI −13.81, 118.42). FOV = 31 μm × 94 μm. ***i***, Top: low magnification image, with white box indicating the location of lower panel image. Scale bar = 50 μm. Bottom: Representative maximum projection of super-resolution image from the superficial dorsal horn laminae showing colocalization of Thy1-GFP-labeled A-fiber (yellow) with presynaptic VGluT1 (magenta) and postsynaptic HOMER (cyan). Scale bar = 5 μm. ***j*,*k***, Super-resolution images of boxed areas in a (bottom panel) with *xy*, *xz*, and *yz* views showing colocalization of Thy1-GFP-labeled A-fiber (yellow), presynaptic VGluT1 (magenta), and postsynaptic HOMER (cyan), as well as surface rendered view on the right. White arrowhead in ***j*** indicates a Thy1-GFP and VGluT1 positive presynaptic bouton without postsynaptic HOMER. Scale bars = 1 μm. Numbers of animals (*n*) as indicated. Black and red data points indicate females and males, respectively. ***l***, Total sum of grey values (intensity) in FOV (295 × 295 μm) for IB4 stain, *p* = 0.97 (Welch's *t* test). ***m***, Representative confocal images of IB4 nonpeptidergic C-fiber terminal innervation in control (left) and *Tmem16f* cKO (right) spinal cord dorsal horn. Scale bar = 50 μm.

Thy1-GFP expression and VGluT1 immunohistochemistry were used to identify myelinated primary afferent terminals in adult *Tmem16f* and control animals (all animals were hemizygous for Cre, controls were nonfloxed. See Materials and methods for details). Neonatal deletion of microglial *Tmem16f* resulted in increased A-fiber terminal occupancy in the superficial dorsal horn in adult animals compared to control mice (Fig. 2*a*,*b*,*d–f*, upper panels). As Thy1-GFP is expressed in only a small number of sensory neurons and varied across animals, all subsequent quantification was performed using VGluT1 immunohistochemistry (Fig. 2*a* upper panels). *Tmem16f* cKO mice had greater primary afferent VGluT1 synaptic density throughout the dorsal horn than control mice as measured by both the total volume and number of puncta (Fig. 2*b*, left and right, respectively) [unpaired mean difference VGluT1 volume, 799.28 µm^3^ (95.00% CI 450.09, 1,136.35); VGluT1 puncta, 533.67 (95.00% CI 356.12, 695.50)] In contrast, the local inhibitory VGAT synapse density was unaltered (Fig. 2*a*,*c*) [unpaired mean difference VGAT volume, −168.31 µm^3^ (95.00% CI −562.61, 253.44); VGAT puncta, −159.19 (95.00% CI −416.42, 148.88)].

In contrast to lamina I/II, the VGluT1 density in lamina III was unaltered (Fig. 2*g*,*h*), suggesting that the changes observed are mainly driven by an increase in VGluT1 terminal projections in superficial laminae only [unpaired mean difference superficial/LI VGluT1 puncta, 201.33 (95.00% CI 110.90, 292.00); deep/LIII VGluT1 puncta, 56.61 (95.00% CI −13.81, 118.42)].

To determine whether the superfluous VGluT1 terminals form synaptic contacts, we co-labeled with the postsynaptic density marker Homer. The colocalization of VGluT1-positive Thy1-GFP-labeled A-fiber terminals with Homer were increased in *Tmem16f* cKO animals, suggesting that a surplus of functional VGluT1-positive synaptic contacts are formed in the superficial dorsal horn of *Tmem16f* cKO animals (Fig. 2*i*–*k*).

Thus, the targeted deletion of microglial *Tmem16f*, and consequently impaired phagocytosis, specifically disrupts the normal developmental pruning of excitatory afferent A-fiber terminals in the superficial dorsal horn. Unmyelinated C-fiber terminals in the dorsal horn were unaffected in *TMEM16f* cKO mice (Fig. 2*l*,*m*) indicating that TMEM16f-mediated pruning is specific to A-fibers in the spinal dorsal horn.

### Neonatal *TMEM16f* deletion in microglia increases behavioral dynamic touch sensitivity in adulthood

**Figure 3.**
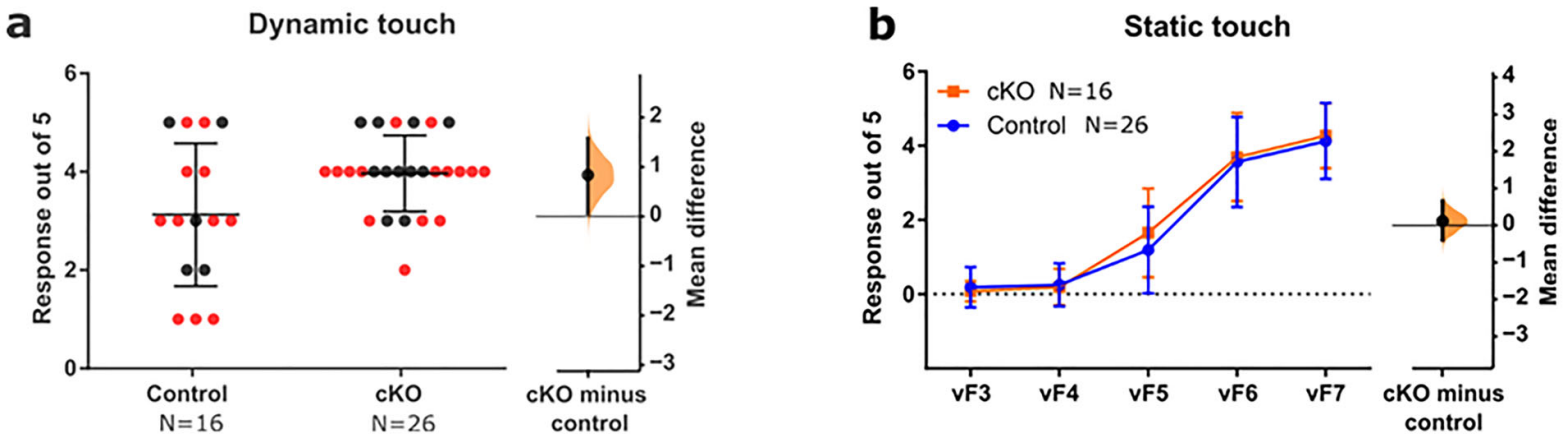
Neonatal *Tmem16f* deletion in microglia increases dynamic touch sensitivity. ***a***, Dynamic touch withdrawal response for *Tmem16f* cKO animals are higher than control animals, mean difference 0.83 (95% CI 0.072, 1.57). ***b***, vF withdrawal response did not differ between *Tmem16f* cKO and control animals, mean difference 0.12 (95% CI −0.39, 0.65) as indicated. Black and red data points indicate females and males, respectively.

With ectopic A-fiber terminals retained in the adult dorsal horn and with evidence of synaptic connectivity, we reasoned that the increase in superficial dorsal horn A-fiber terminals in *Tmem16f* cKO mice would alter behavioral sensitivity to hindpaw tactile stimulation. Cutaneous hindpaw dynamic sensitivity was measured using the number of hindlimb withdrawal reflexes following repeated dynamic touch along the plantar surface of the paw. *Tmem16f* cKO mice showed a significantly greater number of withdrawals in response to dynamic touch than controls [unpaired mean difference 0.83 (95% CI 0.072, 1.57)]. In contrast, static cutaneous sensitivity, measured by application of graded von Frey hair (vF) to the plantar surface was not significantly different in *Tmem16f cKO* and control mice (Fig. 3). *Tmem16f* cKO animals therefore displayed selective cutaneous hypersensitivity to dynamic low-threshold tactile stimulation.

### Dorsal horn sensory neuron electrophysiology reveals expanded dynamic touch receptive fields in adult microglial *Tmem16f* cKO mice

We then asked whether the selective behavioral sensitivity to tactile stimulation as a consequence of excessive A-fiber terminals in dorsal horn is mediated by changes in sensory evoked activity in dorsal horn neurons. To test this, we performed in vivo single unit extracellular recording of wide dynamic range (WDR) neurons in the dorsal horn of anaesthetized *Tmem16f* cKO and control mice. WDR neurons, recorded 200–550 µm below the spinal cord surface, were used because they receive input from all cutaneous sensory afferent types (Aβ, AΔ, and C), project supraspinally via ascending pathways and mediate polysynaptic reflexes (Menetrey et al., 1977; Woolf and King, 1987; You et al., 2003). Changes in their activity therefore predict both sensory output and reflex behavior. Cells were defined as adapting and nonadapting wide dynamic range neurons (WDR) depending on their stimulus response firing properties, as described previously (Lee et al., 2019) (see Materials and methods) and proposed to represent excitatory and inhibitory neurons, respectively. Of the 30 cells recorded from control mice, 8 were nonadapting, and 22 were adapting. Of the 28 cells recorded from *Tmem16f* cKO mice, 5 were nonadapting and 23 were adapting. This is consistent with the expected ratio of 1:2 of inhibitory to excitatory cells as reported in previous studies (Todd, 2010; Abraira et al., 2017; Lee et al., 2019) (*p* = 0.56 for control and *p* = 0.11 for cKO in the binomial test against expected ratio). For each cell recorded, the number of spikes evoked by dynamic and static innocuous touch applied to the plantar hindpaw was quantified. In addition the cutaneous receptive field area was measured by mapping the sensitivity to dynamic touch across the whole plantar surface.

**Figure 4.**
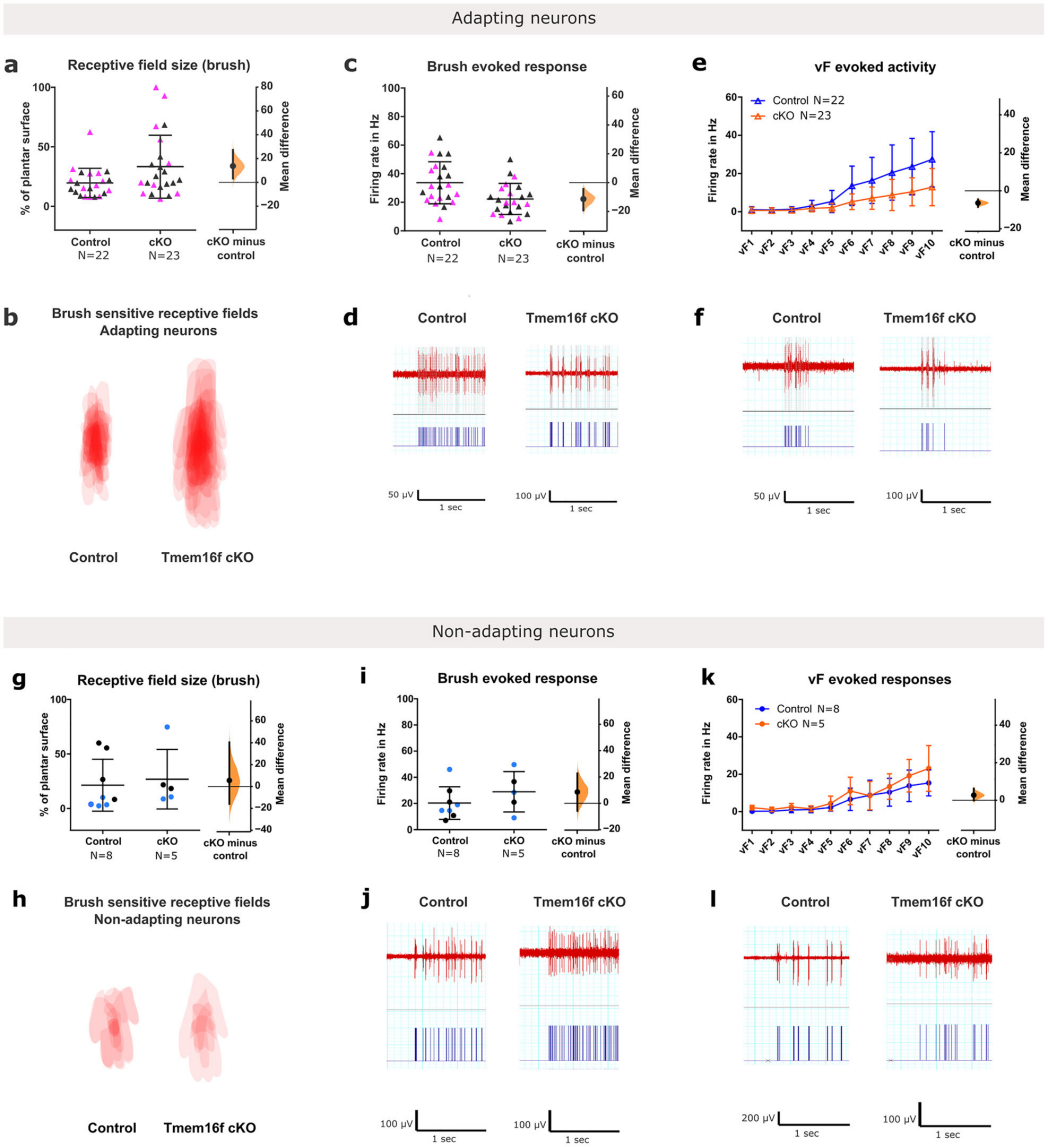
Neonatal *Tmem16f* deletion in microglia increases dynamic receptive field size, but decreases sensitivity to localized dynamic and static stimulation. ***a***, Dynamic touch receptive field size is increased for adapting neurons in *Tmem16f* cKO, mean difference 13.7 (95.00% CI 3.3, 27.2). ***b***, Overlay of receptive fields in shown (***a***). ***c***, Dynamic touch evoked response is decreased for adapting neurons in *Tmem16f* cKO, mean difference −11.4 (95% CI −19.2, −4.5). ***d***, Sample traces of adapting neuron firing in control and cKO animals to dynamic touch stimulation with raster plots underneath. ***e***, Static touch evoked activity was decreased for adapting neurons in *Tmem16f* cKO, mean difference −6.4 (95% CI −8.5, −4.5). ***f***, Example firing trace of an adapting cell from control and cKO animals to vF stimulation with raster plots underneath. ***g***, Dynamic touch receptive field sizes was unchanged for nonadapting neurons in *Tmem16f* cKO, mean difference 5.53 (95.00% CI −15.91, 40.43). ***h***, Overlay of receptive fields shown in (***g***). ***i***, Dynamic touch evoked response was unchanged for nonadapting neurons in *Tmem16f* cKO, mean difference 8.62 [95.00% CI −5.78, 22.72]. ***j***, Example firing trace of nonadapting cell from control and cKO animals to dynamic touch stimulation with raster plots underneath. ***k***, Static touch evoked activity was decreased for nonadapting neurons in *Tmem16f* cKO, mean difference 2.74 [95.00% CI −0.074, 6.25]. ***l***, Example firing trace of a nonadapting cell from control and cKO animals to static touch stimulation with raster plots underneath. Numbers of animals (*n*) as indicated. Black and colorful data points indicate females and males, respectively.

We reasoned that the presence of excessive A-fiber terminals the *Tmem16f* cKO dorsal horn would increase their connectivity with a larger population of postsynaptic neurons in the lumbar cord, leading to individual dorsal horn cells having greater dynamic touch cutaneous receptive field areas. To test this, the borders of dynamic touch receptive fields were mapped across the plantar surface of the hindpaw and the areas of the fields measured. Consistent with increased A-fiber synaptic contacts, the mean dynamic touch receptive field sizes for adapting neurons were increased by almost 50% [unpaired mean difference 13.7 (95.00% CI 3.3, 27.2)], with a notable subpopulation of cKO neurons expanding their receptive fields to cover the entire, or majority of the plantar surface (Fig. 4*a*,*b*). To test whether the expanded dynamic touch receptive fields in *Tmem16f* cKO adapting neurons reflected a generalized increased in excitability to cutaneous stimulation, we quantified the number of spikes evoked in response to localized the dynamic touch and vF stimulation applied within the receptive fields of individual neurons. The number of spikes evoked by defined dynamic and static stimulation within receptive fields was reduced in *Tmem16f* cKO-adapting neurons compared to controls [unpaired mean difference dynamic touch, −11.4 (95% CI −19.2, −4.5); vF, −6.4 (95% CI −8.5, −4.5); Fig. 4*c*–*f*], showing that the neurons in Tmem16f mice are not intrinsically more excitable than in controls.

The same measures were applied to nonadapting neurons. In this population, dynamic touch receptive field sizes did not differ between control and *Tmem16f* cKO mice (Fig. 4*g*–*j*). However, a selective increase in spikes evoked by static touch (but not dynamic touch) was observed in *Tmem16f* cKO animals compared to controls [mean difference 2.74 (95% CI −0.074, 6.25), *F*_(1; 110)_ = 7.014, *p* = 0.0093); Fig. 4*k,l*].

## Discussion

Here, we report that dorsal horn microglia have a distinct phenotype during the first postnatal week characterized by high phagocytic activity, which coincides with postnatal engulfment of A-fiber terminals in the dorsal horn, supporting a role for microglia in developmental dorsal horn remodeling. We further show that a disruption of microglial function by targeted deletion of *Tmem16f* during early postnatal life impairs microglia-mediated A-fiber refinement in the dorsal horn leading to long-term changes in dorsal horn circuit function and reflex behavior that persist into adulthood. Together, our data suggest that microglia-mediated refinement of A-fibers during the early postnatal period is critical to both normal dorsal horn development and appropriate spatial encoding of dynamic touch.

We examined a subset of VGluT1 positive A-fibers, which are predominantly low-threshold, myelinated Aβ mechanoreceptors (Todd et al., 2003; Chamessian et al., 2019), and primarily transduce innocuous mechanical stimulation of the skin. In adults, A- and C-fiber terminals are segregated in the superficial laminae, but during the neonatal period, A-fiber inputs dominate the superficial dorsal horn, as nociceptive C-fiber inputs are weak, forming increasingly strong synaptic connections between P5–10 (Fitzgerald and Gibson, 1984; Baccei et al., 2003). This coincides with lower cutaneous mechanical activation threshold, which together with immature local and descending inhibition underpins the behavioral tactile sensitivity and exaggerated reflex behavior in neonates (Fitzgerald, 1985, 2015; Fitzgerald et al., 1988; Koch and Fitzgerald, 2013).

In the brain microglia have been shown to exhibit a specialized phagocytic phenotype at P4/5 (Hammond et al., 2019), while our data suggest that the peak of microglial phagocytic activity is later in the spinal cord at around P10. Moreover, dorsal horn microglial density observed in our study appears to be higher than in various brain regions studied at comparable ages (Schwarz et al., 2012; Perez-Pouchoulen et al., 2015). Together, this suggests that dorsal horn microglia follow a different developmental trajectory than brain microglia.

We show here that dorsal horn microglia phagocytose A-fiber projections in both the superficial (laminae I–II) and deeper (laminae III–IV) laminae during normal postnatal development with most of the A-fiber engulfment occurring before P10. Pruning is selective for A-fibers generally, not just those with superficially projecting terminals. The lysosomal volume and engulfed fiber volume are increased in both superficial (Fig. 1*b*,*d*) and deeper (Fig. 1*c*,*e*) laminae. This is less evident in the images of deeper laminae as the greater density of A-fibers masks the pruning. However this does not discount the possibility of a dorsoventral gradient in pruning and/or TMEM16f-independent pruning mechanisms. It is important to note that the overall extent of A-fiber engulfment is likely underestimated, as the *Vglut1*-Cre reporter line used here only expresses tdT in a subset of A-fibers (Chamessian et al., 2019). Further, the developmental upregulation of *Vglut1* expression (Minelli et al., 2003; Miyazaki et al., 2003) means that the engulfment will be especially underestimated in younger animals, as their A-fibers might be present but not yet expressing tdT. In the brain, it has been shown that adult levels of VGluT1 protein expression are not achieved until P20 (Minelli et al., 2003). Therefore, the fall in the A-fiber engulfment over the first postnatal week is likely to be steeper than what Figure 1*d*,*e* suggests.

Neonatal *Tmem16f* cKO resulted in an increase in the number of Thy1-GFP-positive VGluT1 synapses in the superficial dorsal horn in adults, compared to controls suggesting that *Tmem16f* function is necessary for microglia-mediated refinement of A-fibers. While the increased density of excitatory synaptic terminals is predominantly in lamina I/II, the density of inhibitory VGAT presynaptic terminals was unchanged from controls in *Tmem16f* cKO animals, suggesting that *Tmem16f* mediated engulfment is specific to both synapse identity and location.

Receptive field sizes of spinal wide dynamic range neurons are larger in neonates and gradually reduce in size as the animal ages (Torsney and Fitzgerald, 2003), as a consequence of both maturing local and descending inhibitory control and refinement of primary afferent input to the dorsal horn (Koch et al., 2012). Furthermore this is an activity-dependent process and can be prevented by chronic NMDA receptor blockade (Beggs et al., 2002). Our results show that the enlarged dynamic touch receptive field sizes of neonatal adapting, putative excitatory spinal neurons (Lee et al., 2019) persist into adulthood in Tmem16f cKO mice. This is unlikely to be due to a generalized increase in excitability or local disinhibition (Zieglgänsberger and Herz, 1971; Takeda et al., 2000) as the evoked activity of the neurons was not increased. The data suggest that it is the lack of activity-dependent refinement of A-fiber terminals in postnatal life that leads to a maintained enlargement of tactile receptive fields into adult life and that this is most evident in response to dynamic rather than static stimulation. During a normal postnatal development, synaptic connections are strengthened or weakened according to the degree of pre-and post-synaptic correlated activity, with poorly correlated, weak connections removed via synaptic pruning. While that correlation of activity is intact in our experiments, it is the agents of that refinement/pruning; phagocytic microglia, which are incapacitated through selective deletion of *Tmem16f* and the ectopic, superficially projecting terminals that would otherwise be eliminated are retained. It should be noted that CX3CR1 is expressed by microglia and also peripheral monocytes and macrophages. Therefore CX3CR1-mediated gene deletion will consequently affect these peripheral cells. Unlike microglia, peripheral immune cells are constantly turning over. Batti et al. (2016) showed that peripheral CX3CR1-expressing macrophage numbers were re-established to control levels by 3 d post-depletion. Therefore it is likely that there is a short period following tamoxifen administration during which peripheral macrophages are reduced and that it is unlikely to impact on the long-term effects described here.

*Tmem16f* cKO mice showed increased behavioral sensitivity to dynamic, but not static, touch. This increase in behavioral sensitivity is likely driven by the increase in dorsal horn excitatory touch receptive field size. These behavioral and electrophysiological results are evidence that increased density of A-fiber terminals in the superficial dorsal horn leads to widespread activation across dorsal horn and reflex networks in response to dynamic touch. The accompanying reduction in dorsal horn evoked spike activity in response to dynamic tactile stimulation may be a consequence of homeostatic synaptic downscaling, a compensatory form of activity-dependent plasticity in which neural circuit stability is maintained by reducing neuronal firing rate when the overall network activity is elevated (Siddoway et al., 2014). The selective increase in static spike activity in inhibitory nonadapting neurons may also contribute to such downscaling.

We conclude that dorsal horn microglia phagocytose A-fiber central terminals during normal postnatal development and that disruption of microglial function can lead to long-term structural and functional changes in the dorsal horn and behavioral responses to dynamic touch. These findings shed light on the normal developmental mechanisms that underlie somatosensory development and identify a key role for microglia in this process.
